# 3D-Printed, Liquid-Filled Capsules of Concentrated and Stabilized Polyphenol Epigallocatechin Gallate, Developed in a Clinical Trial

**DOI:** 10.3390/antiox12020424

**Published:** 2023-02-09

**Authors:** Philippe-Henri Secretan, Victoire Vieillard, Olivier Thirion, Maxime Annereau, Hassane Sadou Yayé, Alain Astier, Muriel Paul, Thibaud Damy, Bernard Do

**Affiliations:** 1 Matériaux et Santé, Université Paris-Saclay, 91400 Orsay, France; 2 Department of Pharmacy, Henri Mondor Hospital, AP-HP, 94000 Créteil, France; 3 Clinical Pharmacy Department, Gustave Roussy Cancer Campus, 94805 Villejuif, France; 4 Department of Pharmacy, Hôpitaux Universitaires Pitié-Salpêtrière, AP-HP, 75013 Paris, France; 5 EpidermE, University Paris Est Creteil, 94010 Creteil, France; 6 Department of Cardiology, Henri Mondor Hospital, AP-HP, 94000 Créteil, France

**Keywords:** epigallocatechin gallate, formulation, eutectic solution, stability, bioavailability, oral solution

## Abstract

In vitro studies have shown that epigallocatechin gallate (EGCG), the most potent antioxidant of the green tea polyphenol catechins, is able to effectively prevent the formation of amyloid plaques and induce their clearance. However, its high chemical reactivity promotes high chemical instability, which represents a major obstacle for the development of pharmaceutical forms containing solubilized EGCG, an essential condition for a better systemic passage via the oral route. After discovering that EGCG forms a deep eutectic with choline chloride, we exploited this property to formulate and patent liquid-filled capsules containing 200–800 mg of soluble EGCG in easy-to-administer sizes. The gelatin envelopes used are of the conventional type and their filling has been achieved using 3D printing technology. Not only did the EGCG-choline complex allow the formulation of hydrophilic solutions with a high concentration of active substance but it also contributed significantly to its chemical stability, since after at least 18 months of storage at 25 °C/60% RH and one year at 40 °C/75% RH, the capsules show unchanged hardness, chromatographic profiles and antioxidant activity compared to T0. Preclinical studies in monkeys showed that bioavailability was increased by a factor of 10 compared to marketed capsules comprising EGCG powder. This pharmaceutical development was conducted in the context of upcoming clinical trials to evaluate EGCG alone or in combination when treating transthyretin and light-chain cardiac amyloidosis.

## 1. Introduction

The extracellular deposition of misfolded proteins in organs is responsible for various disorders, such as transthyretin cardiac amyloidosis, a disease causing an infiltrative/restrictive cardiomyopathy [1]. To date, diagnosing and treating transthyretin cardiac amyloidosis remains challenging [2]. Among potential therapeutic agents to treat transthyretin cardiac amyloidosis, Epigallocatechin-gallate (EGCG), a major polyphenol of green tea, could be of therapeutic interest, as EGCG is able to inhibit transthyretin amyloid fibril formation and disaggregate amyloid deposits [3]. This potential therapeutic interest is supported by two-open label observational studies [4,5] where 12 months of green tea consumption reduced the left ventricular mass by 6–13%.

Bioavailability determinations of catechins have shown that the levels of detected epicatechins (EC) and epicatechin-3-gallate (ECG) are too low to be of any therapeutic value. Hence, most research considers mostly, EGCG and to a lesser extent EGC [6]. Still, after oral administration, the bioavailability of EGCG was found to be very low in humans, resulting in plasma concentrations of 5 to 50 times less than the concentration shown to exert biological activities in vitro systems [7]. To increase the amount of absorbed EGCG, a method for producing O-α-glucoside derivatives of phenolic compounds (such as EGCG) by incubating sucrose and a glucansucrase with the phenolic compounds was proposed [8]. However, this method involves structural changes of EGCG, which may impair its properties. Other research using EGCG and nanoparticles has been carried out. These include: coating a nanoparticle, such as gold, with EGCG; the use of encapsulated EGCG in nanoparticles along with anti-cancer drugs; outer ligands that bind to specific targets; or outer polymers that will enhance the intestinal absorption of EGCG [9,10]. However, changes in release rates in the gastrointestinal tract induced by the use of nanoparticles could make the EGCG biopharmaceutical phase even more complex and far less predictable.

In addition to the kinetic and biopharmaceutical aspects, EGCG powder has the disadvantage of being very powdery and therefore spreads fine particles in the air during the mixing with excipients and capsule filling steps, which makes the cleaning of the manufacturing areas very difficult, leading to a high risk of cross-contamination, especially as the doses of EGCG per therapeutic unit are high (200–600 mg).

For all these reasons, we opted for the development of an oral liquid form, provided that the EGCG could be chemically stabilized. However, due to the very strong bitterness of a concentrated EGCG solution, it was necessary to try to mask the taste by encapsulating the liquid without altering the integrity of the gelatin envelope over time.

Therefore, to afford the possibility of performing clinical trials where EGCG’s full potential is challenged, there is still a need to provide an EGCG composition containing an elevated amount of EGCG and enabling satisfactory stability and bioavailability. This manuscript describes the formulation of capsules composed of a liquid eutectic system of EGCG, which comprises EGCG, polyethylene glycol PEG 400, PEG 4000, water and a choline salt. To support the use of this formulation in a clinical trial aiming at treating light chain and transthyretin cardiac amyloidosis, the quality performances of the capsules and bioavailability of EGCG eutectic solution have been assessed. However, only some of the data are presented, enough to show the pre-clinical performance of the developed system.

## 2. Materials and Methods

### 2.1. Materials and Reagents

Green tea extract, containing 97% EGCG (and 3% ECG) is considered as an isolated substance, chemically defined according to the Reflection Paper on the Level of Purification of Extracts to be Considered as Herbal Preparations EMA/HMPC/186645/2008 [11]. As it is food grade, it was supplied by Sunfull Bio-tech Co., Ltd (Changsha, China). Therefore, for simplicity, the raw material within the manuscript is directly referred to as “EGCG”. All the excipients used in the product composition were of pharmaceutical grade. Polyethylene glycol 400 and polyethylene glycol 4000 were purchased from Inresa (Bartenheim, France), whereas choline chloride was acquired from Sigma-Aldrich (Saint-Quentin-Fallavier, France). Capsugel PCcaps^®^ (tiny gelatin capsules ideal for oral delivery of the isolated active component in pre-clinical animal studies) were purchased from Lonza (Bâle, Switzerland).

The reagents, 2,2-azino-bis(3-ethylbenzothiazoline-6-sulfonic acid) diammonium salt (ABTS), Trolox (6-hydroxy-2,5,7,8-tetramethylchroman-2-carboxylicacid) and potassium persulfate were purchased from Sigma-Aldrich (Saint-Quentin-Fallavier, France). The analytical grade solvents were obtained from Merck (Fontenay-sous-Bois, France).

### 2.2. Manufacturing Protocol

The preparation consisted of hard-gel capsules containing 400 mg of EGCG in solution, packaged in a 250 mL high-density polyethylene bottle, closed with a cap with a polyethylene seal. The composition of the solution for capsule filling and the function of each component are given in Table 1. The EGCG solution was prepared by dissolving the ingredients in purified water.

The filling step was performed by use of a 3D printer that features several print heads that can function independently or simultaneously (Medimaker 2, supplier FabRx–29-39 Brunswick Square UCL, London WC1N 1AX). This operation requires a head that allows liquid deposition by extrusion at 37 °C. This process enables the depositing of the EGCG solution with semi-solid technology (Injeckt^®^ syringes from Becton Dickinson, Rungis, France).

The right amount of EGCG solution deposited is obtained by calibration associated with the diameter of the extrusion head, the pressure exerted by the piston of the syringe, and the motor steps.

The printer then carries out these deposits according to a pre-established plan by means of computer-assisted software. To avoid any possibility of dripping, the pressure is systematically released after each extrusion.

### 2.3. Stability Testing

To assess whether the eutectic system could help to protect EGCG from degradation, 1 mL of the reference standard suspension in water (EGCG at a concentration of 26.7 mg·mL^−1^) and 1 mL of the formulation were exposed to alkali, thermal and photolytic stress, according to the conditions detailed in the Supplementary Materials (Table S1). At the end of exposure, the solutions were diluted at 1/100 in distilled water and analyzed by high-performance liquid chromatography (HPLC).

Long-term stability studies were carried out as per the International Conference on Harmonization ICH Q1A(R2) [12]. The protocol applied is given in the Supplementary Materials.

The content (%) of each impurity or degradation product was determined according to the area normalization method through the following equation:

(1) $$\mathrm{Content}\;\mathrm{of}\;\mathrm{impurity}\;\left(\%\right)=\frac{A\left(u\right)}{A\left(t\right)}\times 100,$$

where A(u) is the peak area of the corresponding impurity from the sample solution and A(t) is the total peak area of the sample solution.

### 2.4. Analytical Conditions

#### 2.4.1. Chromatographic Conditions

The chromatographic conditions for the drug assay in the stability study are detailed in the Supplementary Materials.

The chromatographic conditions for drug assay in the blood were based on those proposed by Lourdes et al. [13]. The molecular and daughter ions were selected for each molecule after direct infusion into the MS–MS system. The analytical method consisted of the precipitation of the proteins by using methanol followed by liquid chromatography (LC) coupled to tandem mass spectrometry (MS/MS) analysis. According to the expected sensitivity, at least eight calibration standards were used to prepare the calibration curve in plasma. The corresponding correlation coefficient was calculated and had to be higher than 0.75 for the continuation of the in vivo test. The calibration range tested was 4 to 5000 ng·mL^−1^ of EGCG in plasma. The difference between the mean concentration observed and the nominal concentration was used to estimate the deviation of the method.

#### 2.4.2. Mass Spectrometry Conditions

The MS characterization of the EGCG–choline chloride interaction was performed by directly infusing the solutions in an LTQ-Orbitrap Velos Pro system (Thermo Fisher Scientific, Waltham, MA, USA). Electrospray ionization in negative ion mode (ESI^−^) was chosen as a source. The source voltage, the source and the capillary temperatures were fixed at 3.02 kV, 53.9 °C and 300 °C, respectively. Xcalibur^®^ software (version 2.2 SP 1.48) was used to process MS data.

### 2.5. In Vitro Dissolution Test

The tests were performed using a paddle method on a Sotax apparatus. pH 1.2 and pH 6.8 dissolution media were prepared according to the current European Pharmacopoeia. The volume of dissolution media, the paddle rotation speed, the optical path length in the ultraviolet (UV) reading cells and the detection wavelength were 900 mL, 75 rpm, 0.2 mm and 275 nm, respectively. The dissolution percentage was calculated in relation to calibration solutions ranging from 0.6 to 1.0 µg·mL^−1^.

### 2.6. Antioxidant Activity Assay

Antioxidant activity was assayed by measuring oxygen radical absorbance capacity (ORAC assay). The assay was performed based on a protocol described by Al-Duais et al. [14]. A mixture of acetonitrile and water (50/50) was used as dissolving agent and diluent. ABTS^•+^ was obtained after 16 h incubation of 7 mM ABTS with 245 mM potassium persulfate in the dark at room temperature. The solution was then diluted to reach an absorbance of about 0.7 at 734 nm. Several Trolox solutions at concentrations from 12.5 to 400 µM and three sample solutions (final concentration 0.25 µM) were prepared.

A total of 10 μL of each Trolox standard solution and each sample solution were administered into separate microtiter plate wells in triplicate. Then 190 µL of the ABTS^•+^ solution prepared as above were added into each well. The solutions were then shaken, and the microplate was read at 734 nm after 5 min.

### 2.7. Bioavailability Studies

One capsule of green tea extract (containing 97% of EGCG), was administered to each of the three Cynomolgus monkeys using a specific device to place the item on the back of the tongue. The contents of green tea extract in tested capsules were adapted according to the monkey’s weight to ensure an administration of 27 mg EGCG per kg of monkey weight.

Then, a washout period of seven days was applied.

EGCG green tea extract solution, formulated as per Table 1, was orally administered to three Cynomolgus monkeys using a gastro-esophageal tube or a nasogastric tube and an appropriate syringe. After administration, the tube was rinsed with 5 mL of water and flushed with 5 mL of air to ensure that the entire dose (27 mg EGCG per kg of monkey weight) was delivered.

At each sampling time, 0.5 mL of blood was collected from the femoral or cephalic vein. The samples were centrifugated (2500× *g* for 10 min at 5 °C), aliquoted, shipped and stored at −80 °C until analysis. The analytical conditions for EGCG quantification in the serum sample are reported in Section 2.4.1.

The quantities of EGCG administered per animal and sampling times are reported in Table 2.

### 2.8. Statistical Analysis

All statistical tests were performed using GraphPad Prism V.9.5.0. for Windows (GraphPad Software, La Jolla, CA, USA). Unpaired *t*-tests were used to compare the mean of Trolox equivalent obtained for pairs of the three groups: “green tea extract powder”, “extemporaneous capsules” and “capsules stored 1 year at 40 °C”.

## 3. Results and Discussion

### 3.1. Formulation Strategy

Our formulation strategy resulted from the simultaneous consideration of (i) clinical and methodological objectives, (ii) regulatory and technical constraints and (iii) opportunities to deploy know-how combining chemistry and cutting-edge pharmaceutical technologies.

The planned dosing regimen consists of administering EGCG alone or in combination at a dose of 400 mg twice daily. In all cases, the study was a double-blind trial versus placebo.

Apart from Veregen^®^ (an American pharmaceutical product), an ointment containing 10% green tea extract to treat anal-genital warts in AIDS (human immunodeficiency virus) patients, the marketing of which was suspended in 2017, there has been no other medicinal product based on green tea extract or isolated EGCG. As a pharmaceutical herb, green tea has a monograph in the European Pharmacopoeia (monograph 1668), but its wide content range and use do not meet the needs of clinical trials in terms of dose control, ease of administration and blinding requirements. Many capsules containing green tea extract powder or isolated EGCG powder, with unit contents ranging from 50 to 350 mg, are marketed as dietary supplements. However, given the content of the active substance administered to the patient in the clinical trial, the competent authorities questioned were reluctant to authorize the use of the latter without mandatory pharmaceutical control guaranteeing the quality and safety of the products.

**Figure 1:**
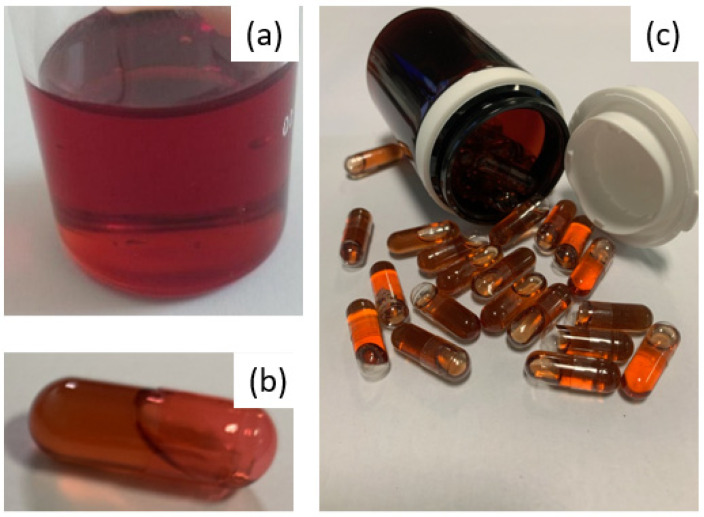
(**a**): appearance of the fill before division into capsules. (**b**): capsule comprising 400 of EGCG. (**c**): typical containers for one month’s treatment.

For us, as a hospital structure, this was a challenge to meet, in terms of the control of the active substance used and the design of a product, the process of which needed to be simple and robust in order to overcome the constraints linked to a significant need for treatment units throughout the clinical trial and the difficulty of handling the very powdery active substance, knowing that 400 mg of EGCG are required per dose. Indeed, the first tests of the mixing and distribution of powders in capsules demonstrated the need to use at least capsule size 00 and, above all, revealed a significant risk of cross-contamination related to the strong adhesion of the fine EGCG powder, making the cleaning of surfaces difficult. To avoid the problems associated with EGCG powder, a good way to proceed was to develop a liquid-based formulation, while having to mask the particularly astringent and bitter taste of EGCG, which can be an obstacle to both patient adherence to the treatment and successful blinding. This represented the main challenge regarding the manufacture of capsules containing 400 mg of EGCG in solution, under the condition of having significantly improved the solubility and chemical stability [10] of this polyphenol. Indeed, the solubility tests of isolated EGCG that were performed at 25 °C show a saturated aqueous solution at 14.6 mg·mL^−1^ and solutions in PEG 400 or propylene glycol saturated at less than 5 mg·mL^−1^. To solubilize at least 43% (*w*/*w*) EGCG in a hydrophilic liquid blend, we formulated a proprietary liquid-based filler where the deep eutectic EGCG/choline chloride or EGCG/choline bitartarate (PCT/EP2020/064876) is diluted in a PEG400/PEG4000/water mixture. The resulting system is a dense and viscous red solution (Figure 1a), encapsulated in colored but transparent hard gel capsules (Figure 1b) allowing us to guarantee (i) the blinding requirement without having to formulate an equivalent color placebo solution and (ii) the visual examination of contents during stability studies. Due to its high density and viscosity, the repeatable and accurate distribution of the solution in the capsules was achieved using a 3D printer using a semi-solid extrusion.

### 3.2. Filling Solution Characteristics

#### 3.2.1. MS Characterization of the EGCG–Choline Chloride Interaction

As a powerful method for studying non-covalent complexes between the ‘‘host” and “guest”, electrospray ionization mass spectrometry (ESI-MS) [15] was used to highlight EGCG–choline affinity. The solution, the composition of which is given in Table 1, was diluted 1/100 in an aqueous solution of formic acid (0.1%) and directly infused into the source of the electrospray (ESI) of the mass spectrometer.

**Figure 2:**
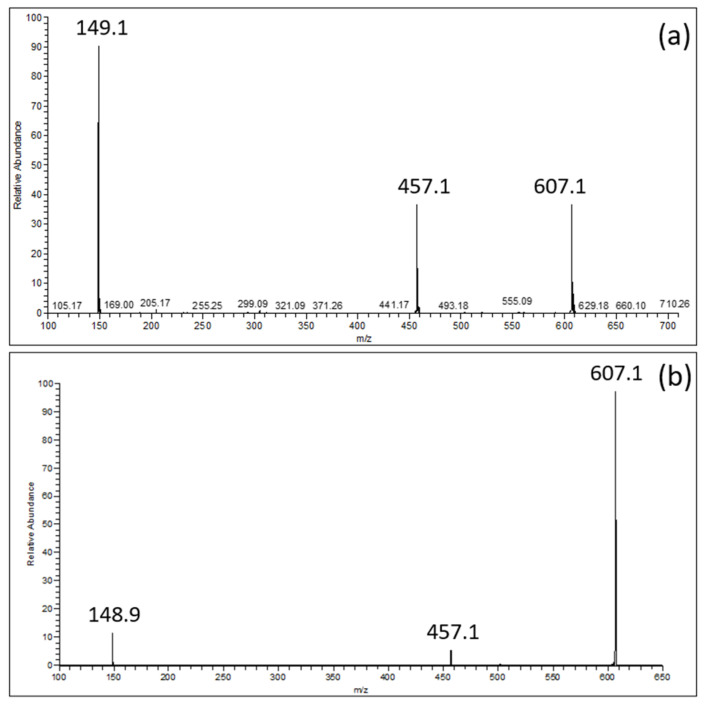
(**a**): ESI-MS spectrum of an EGCG–choline chloride solution; (**b**): ESI-MS^2^ spectrum of the [(EGCG + Choline + HCOOH) − H]^−^ product ion (*m*/*z* = 607.1).

MS spectra comprise not only the deprotonated ion of EGCG ([EGCG − H]^−^; *m*/*z* = 457.1) and that of the choline–formic acid adduct ([(Choline + HCOOH) − H]^−^; *m*/*z* = 149.1) with the relative abundances of about 50% and 100%, respectively (Figure 2a), but also the ion ascribable to the equimolecular EGCG–choline complex ([(EGCG + Choline + HCOOH) − H]^−^; *m*/*z* = 607.1). The EGCG–choline chloride interaction was confirmed by the persistence of the ion representing the EGCG–choline complex (Figure 2b), despite the application of a collision energy of 21 arbitrary units for pulsed Q collision-induced dissociation.

#### 3.2.2. Chemical Stability of the Filling Solution with Regards to Light Irradiation and Temperature

The “isolated substance” of EGCG used in the formulation initially comprises 97% EGCG and 3% ECG. All other catechins and/or components of green tea according to the Green Tea Monograph (Monograph 2668, Eur. Ph.), such as (+)-gallocatechin (GC), (−)-epigallocatechin (EGC), (+)-catechin, (−)-epicatechin (EC) and (−)-gallocatechin-3-*O*-gallate (GCG), may also be present but in this case would have been far below 0.05% (declaration threshold) because they were not detected. Nevertheless, it should be noted that the chromatographic method (see Section 2) that we used to follow any evolution of the isolated substance EGCG in the product is capable of separating and highlighting most of the natural components of the green tea if they appeared throughout the formulation and stability studies, as shown by the chromatogram obtained after the analysis of the USP reference standard decaffeinated green tea extract powder (see Supplemental Materials).

Today, it is well established that the aforementioned catechin derivatives can easily be oxidised and transformed into novel dimeric, oligomeric and polymeric compounds such as theacitrins, theaflavins, theasinensins and theanaphthoquinones, and are also present, to a small extent, in the USP powdered, decaffeinated green tea extract reference standard [16,17,18]. From a safety perspective, these represent the most abundant group of phenolic pigments found in black tea, making up approximately 60% of the solids of a typical black tea infusion, widely consumed around the world and therefore known to be non-toxic. Moreover, the latter are ubiquitous in food chemistry (complex polyphenols from tea and cocoa, aged red wines, non-phenolic components in Maillard reactions, etc.), biological systems and environmental samples, such as waste. At high concentrations, it has been reported that the oxidation of catechins gives way to a reversible trans-epimerization and that the trans-epimers do not have more toxic effects because they possess biological activities similar to those of their cis homologs [19,20]. Moreover, catechin epimerization can be reversible [20,21].

For our research presented in this and the following sections, even if the degradation of EGCG and ECG does not entail the risk of toxicity per se, we nevertheless considered that any product initially present (apart from those assigned to the excipients and other than EGCG and ECG) to be a degradation product which required identification if it appeared in quantities of more than 0.2%, determined according to the area normalization method (see Section 2). After exposing the filling solution, the composition of which is presented in Table 1, to 60 °C for 72 h or to the photolysis conditions described in ICH Q1B for the same period of time, in both cases we observed no color change, demonstrating that, potentially, no significant oxidation of the solution had occurred. On the other hand, on the chromatographic plots, in both cases, the same additional compounds appeared at a relative retention time of 1.1 min and at a content exceeding 0.2%. Subjected to analysis by LC-MS, this compound presents an MS spectrum in all respects comparable to that of EGCG acquired under the same experimental conditions, which, in a perfectly plausible way, led us to attribute it to a trans-epimer compound of EGCG, confirming in passing the fact that at high concentrations (millimolar EGCG concentrations and higher) [20,21], EGCG is more subject to reversible epimerization than to oxidation. The low content of the epimer formed was also a good reflection of the stability of the filling solution, and this was amplified by a formulation where EGCG is fully solubilized at 43.6% (*w*/*w*).

### 3.3. EGCG Capsules Characeteristics

#### 3.3.1. Dissolution Studies

After filling, the dissolution of the capsules was tested at pH 1.2 and 6.8 using the paddle method (75 rpm). The required dissolution media were prepared according to the current Eur. Ph. monograph recommendations on dissolution testing (monograph 51701). The dissolution profiles were carried out on 6 units without ballast. Although all the excipients used are water-soluble, it was critical to ensure that the formulation allowed immediate release, and this was also taken into consideration regarding the expiration duration (see the stability section).

**Figure 3:**
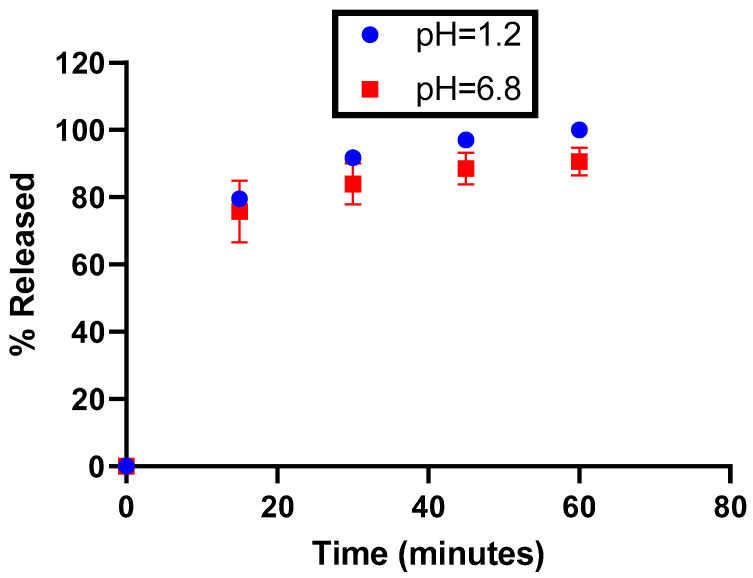
Release profiles of EGCG as a function of the pH.

According to the dissolution profiles shown in Figure 3, the dissolution rate of the capsules is higher in acidic media. However, regardless of the medium tested, the dissolution rate is systematically superior to 75% at 30 min, as well as being reproducible (maximum relative standard deviation = 2.4%), meaning that EGCG is released from capsules at a rate that should enable the complete and reproducible liberation of EGCG in the gastrointestinal tract.

#### 3.3.2. Formal Stability Studies of the EGCG Capsules

The performance of the formulation was also assessed with good hindsight by applying the ICH Q1A(R2) [12] conditions assessing the stability of the capsules present in their primary packaging (see Figure 1c) under accelerated conditions (40 °C/75% RH) and standard (25 °C/60% RH) storage conditions. From these data, an expiry date could be established in order to guarantee the maintenance of the quality of the product during the course of the clinical trial. To date, stability studies are still ongoing and the updated data are discussed here, representing 18 months in standard conditions and 9 months in accelerated conditions. Considering the particular characteristics of the hard gel capsules containing a liquid filling loaded with dissolved EGCG, the monitoring criteria include: the verification of the physical/macroscopic integrity of capsules, EGCG assay, impurity profile and in vitro dissolution. In addition, to ensure that the formulation had not lost any of its antioxidant activity, the oxygen radical absorbance capacity (ORAC) was measured at various times.

The potential leaking of the capsules was investigated by shaking two capsules placed in Falcon tubes with an electric rotator spinning at 100 rpm for 0.5 h. After shaking, the capsules were examined visually by touch and weighed. So far, at each test undertaken throughout the stability study, even after 12-month storage in accelerated conditions, no leaks have been detected and the texture and integrity of the capsules have not changed. This satisfactory but somewhat unexpected result may be due to the high density of the filling solution (approximately 1.25 (20 °C)), which minimizes the interaction of the contents with its envelope.

In the case of dissolution, for the different stability times other than time 0, we did not carry out dissolution profiles but utilized only one dissolution point at 45 min at the most unfavorable pH, namely pH 6.8. Indeed, the objective was to ensure that in the worst case, the capsule still exhibited dissolution in accordance with the specification of the European Pharmacopoeia for an immediate release form. Hence, after 18 months at 25 °C/60% RH and 12 months at 40 °C/75% RH, the EGCG dissolution rates were 86.1% and 87.2%, respectively.

**Figure 4:**
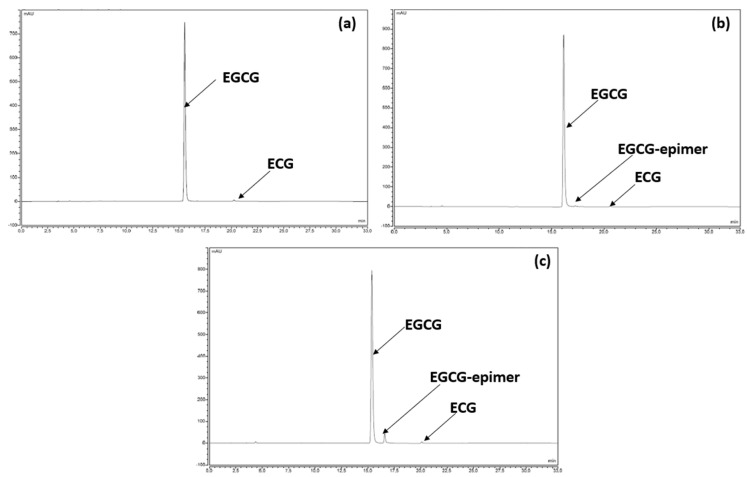
(**a**) Chromatographic profile of the formulation at time 0. (**b**) Chromatographic profile of the formulation after 18 months under standard conditions. (**c**) Chromatographic profile of the formulation after 6 months under accelerated conditions.

In terms of chemical stability, we observed a perfect correlation with the preliminary results presented and discussed in Section 3.2.2. For example, the chromatographic profile after the analysis results of the samples stored for 12 months at 25 °C/60% RH are identical to those recorded at time 0, in the sense that no compound other than EGCG and ECG was detected (Figure 4a). After 18 months under standard conditions (Figure 4b), another compound had appeared but was detected to still be under 0.2%. Under the accelerated conditions after 6 months of storage, the same degradation product was detected at a higher level and continued to increase with time, reaching about 5% (Figure 4c). However, in both cases, the degradation product was none other than the same compound detected and tentatively identified in Section 3.2.2 as stemming from EGCG epimerization.

**Figure 5:**
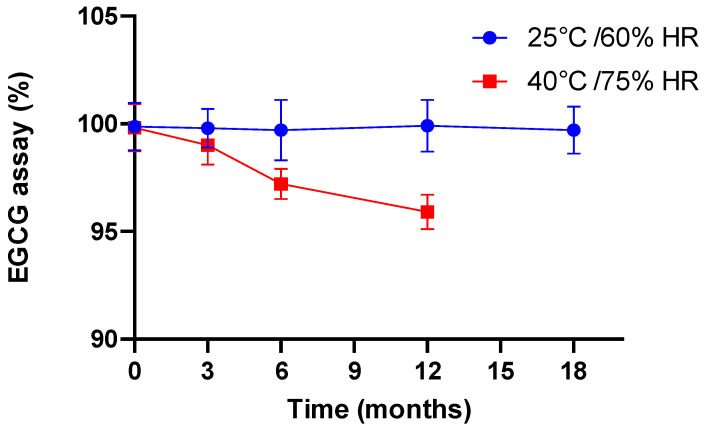
Evolution of EGCG (assay, % with respect to time 0) as a function of time and storage conditions.

These data, in all respects, correlate to the evolution of EGCG content with time, determined with regard to time 0, as shown in Figure 5.

##### Antioxidant Activity

The absence of the change of EGCG antioxidant activity was investigated by performing ORAC assays. Indeed, the ORAC assay has previously been established as a standard method for assessing the activity of hydrophilic antioxidants.

**Figure 6:**
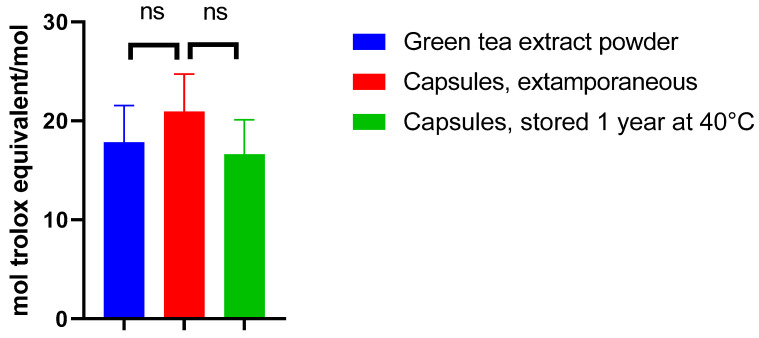
Antioxidant activity expressed in the Trolox equivalent of green tea extract powder of capsules prepared extemporaneously and of capsules stored at 40 °C.

Appropriate amounts of the formulation were taken from extemporaneously prepared capsules and capsules stored at 40 °C for one year. The activity of the corresponding quantities of EGCG was compared to that of the green tea extract powder (containing 97% of EGCG, *w*/*w*) used to prepare the capsules (Figure 6).

When assessing the antioxidant activity of the EGCG powder, the antioxidant activity value (expressed in mol equivalent Trolox/mol) was 17.8, similar to that observed in another study [22]. Incorporating EGCG in this form of dosage did not significantly alter its antioxidant activity immediately after preparation as compared to green tea extract powder (*p* = 0.3688) and after one year of storage at 40 °C as compared to extemporaneously prepared capsules (*p* = 0.2197) (Figure 6).

Based on these results, the proposed formulation should keep its antioxidant potency, even in the long term. This result is in line with the potential good efficacy of the treatment, provided EGCG is absorbed. This latest aspect is investigated in the next section (Section 3.4).

### 3.4. Bioavailability Compared between the Formulation and a Capsule Filled with EGCG Powder

While the stability and dissolution tests of the formulation demonstrated that the developed oral solution had the quality performance necessary for clinical trials, it was necessary to assess whether it had satisfactory biopharmaceutical properties after oral administration. Indeed, the low bioavailability of EGCG results from its extensive degradation in the gastrointestinal tract [23] and its active efflux by efflux pumps, including the multidrug resistance-associated protein (MRP) efflux pumps [24] and the P-glycoprotein [25].

Thus, the performance of the formulation in terms of bioavailability was assessed by administering *per os* (by gavage) to Cynomolgus monkeys (*n* = 3) 27 mg·kg^−1^ of EGCG by use of the formulation, as depicted in the Table 1. The results were compared to those obtained using capsules containing green tea extract (*n* = 3), prepared to afford 27 mg·kg^−1^ of EGCG. The choice of this animal model was based on the fact that Cynomolgus monkeys, as a non-human primate model, are widely used for the prediction of drug absorption in humans and that this animal model displays similar orders of magnitude of expression of apical drug efflux transporters [26].

**Figure 7:**
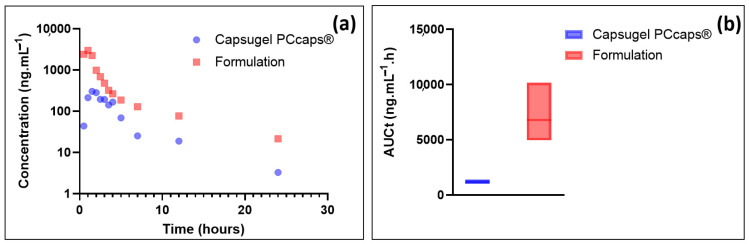
(**a**) Plasmatic concentrations of EGCG as a function of time; (**b**) box plots of the total area under curve.

EGCG plasma concentration and the total area under curve (AUCt) results are depicted in Figure 7.

As these results show, EGCG plasma concentration resulting from the administration of the formulation is, at equal doses administered, much higher than that resulting from the administration of a capsule containing the same amount of EGCG in solid form. Precisely, the data presented in Figure 6 show the potential value of the developed formulation as Cmax (mean = 2996 ng·mL^−1^) and AUCt (6780.7 ng·mL^−1^·h) are, respectively, about 10 and 6 higher than when using Capsugel PCcaps^®^ containing the same amount of EGCG (Cmax = 303 ng·mL^−1^; AUCt = 1154.7 ng·mL^−1^·h).

Based on these results, the formulation greatly enhances the bioavailability of epigallocatechin gallate. This result may be partly explained by the increase in EGCG in vivo stability thanks to the eutectic system. The high concentration of EGCG in the formulation may also have contributed to this increase as it has been shown that the aqueous solubility of drugs is frequently predictive of their bioavailability [27].

Finally, the high increase in EGCG in the gastrointestinal tract of Cynomolgus monkeys may be the consequence of the inhibition of the activity of the efflux protein P-GP, based on the fact that polyphenols are known to exert such effects [25].

## 4. Conclusions

Many recent studies, prospective and retrospective, have shown the tremendous therapeutic potential of EGCG in many fields. Despite this, the latter continues to be marketed only as a dietary supplement taken orally, mainly present in the form of powders put into capsules, while the advantages of liquid dosage forms in terms of bioavailability had been reported for EGCG. However, in soluble form, it is also true that EGCG is particularly unstable regarding oxidation, which is a major obstacle to any development of commercial liquid form and even more so if we want to consider its use as drug.

For the very first time, a formulation strategy based on soluble EGCG is proposed. It consists of capsules containing a concentrated solution of EGCG dispensed using 3D printing technology. An extremely high concentration of EGCG (43.6%, *w*/*w*) in the filling solution enabled the development of a stable drug candidate with improved bioavailability, according to preliminary studies in monkeys, and which meets the quality requirements for clinical trials, which, in this case, concerns cardiac transthyretin and light chain amyloidosis.

## Figures and Tables

**Table 1:** Composition of the solution for capsule filling.

Component	Percentage Formula	Functions
Green tea extract	44.92 (i.e., 43.6% of pure EGCG, *w*/*w*)	Active ingredient
Polyethylene glycol 400	20.29	Solvent and thickening agent
Polyethylene glycol 4000	0.67	Thickening agent
Choline chloride	16.16	Solubilizing agent
Purified water	17.96	Solvent

**Table 2:** Conditions of the bioavailability studies.

Period	Formulation	Target Dose	Quantity of Green Tea Extract (Containing 97% of EGCG) to Administer per Animal	Blood Sampling Times for Analysis
1	Green tea extract capsule	27 mg·kg^−1^	Monkey 1: one capsule at 145.8 mg of green tea extract	30 min, 1 h, 1.5 h, 2 h, 2.5 h, 3 h, 3.5 h, 4 h, 5 h, 7 h, 12 h, 24 h
			Monkey 2: one capsule at 116.1 mg of green tea extract	
			Monkey 3: one capsule at 108.0 mg of green tea extract	
2	Wash-out period (7 days)			
3	Green tea extract eutectic solution (44.92% *w*/*w*)	27 mg·kg^−1^	Monkey 1: 323 mg of eutectic solution (145 mg of green tea extract)	30 min, 1 h, 1.5 h, 2 h, 2.5 h, 3 h, 3.5 h, 4 h, 5 h, 7 h, 12 h, 24 h
			Monkey 2: 258 mg of eutectic solution (116 mg of green tea extract)	
			Monkey 3: 240 mg of eutectic solution (108 mg of green tea extract)	

## Data Availability

Not applicable.

## Supplementary Materials

The following supporting information can be downloaded at: https://www.mdpi.com/article/10.3390/antiox12020424/s1, Figure S1: Chromatogram from analysis of ‘USP powdered decaffeinated green tea extract’ reference standard; Table S1: Stress conditions to assess EGCG stability; Table S2: Sampling and storage protocol for primary batch studies; Table S3: Gradient conditions

## References

[1] Martinez-Naharro A., Hawkins P.N., Fontana M. (2018). Cardiac Amyloidosis. Clin. Med..

[2] Yamamoto H., Yokochi T. (2019). Transthyretin Cardiac Amyloidosis: An Update on Diagnosis and Treatment. ESC Heart Fail..

[3] Ferreira N., Saraiva M.J., Almeida M.R. (2012). Epigallocatechin-3-Gallate as a Potential Therapeutic Drug for TTR-Related Amyloidosis: “In Vivo” Evidence from FAP Mice Models. PLoS ONE.

[4] aus dem Siepen F., Bauer R., Aurich M., Buss S., Steen H., Altland K., Kristen A., Katus H.A. (2015). Green Tea Extract as a Treatment for Patients with Wild-Type Transthyretin Amyloidosis: An Observational Study. Drug Des. Dev. Ther..

[5] Kristen A.V., Lehrke S., Buss S., Mereles D., Steen H., Ehlermann P., Hardt S., Giannitsis E., Schreiner R., Haberkorn U. (2012). Green Tea Halts Progression of Cardiac Transthyretin Amyloidosis: An Observational Report. Clin. Res. Cardiol..

[6] Reygaert W.C. (2014). The Antimicrobial Possibilities of Green Tea. Front. Microbiol..

[7] Chow H.-H.S., Hakim I.A., Vining D.R., Crowell J.A., Ranger-Moore J., Chew W.M., Celaya C.A., Rodney S.R., Hara Y., Alberts D.S. (2005). Effects of Dosing Condition on the Oral Bioavailability of Green Tea Catechins after Single-Dose Administration of Polyphenon E in Healthy Individuals. Clin. Cancer Res..

[8] Auriol D., Nalin R., Robe P., Lefevre F., WO2007144368A3 Water Soluble Phenolics Derivatives with Dermocosmetic and Therapeutic Applications; Filed 13 June 2007. and Issued 18 June 2009. https://patents.google.com/patent/WO2007144368A3/en.

[9] Granja A., Pinheiro M., Reis S. (2016). Epigallocatechin Gallate Nanodelivery Systems for Cancer Therapy. Nutrients.

[10] Krupkova O., Ferguson S.J., Wuertz-Kozak K. (2016). Stability of (−)-Epigallocatechin Gallate and Its Activity in Liquid Formulations and Delivery Systems. J. Nutr. Biochem..

[11] EMA Level of Purification Extracts to Be Considered as Herbal Preparations—Scientific Guideline. https://www.ema.europa.eu/en/level-purification-extracts-be-considered-herbal-preparations-scientific-guideline.

[12] EMA ICH Q1A (R2) Stability Testing of New Drug Substances and Products—Scientific Guideline. https://www.ema.europa.eu/en/ich-q1a-r2-stability-testing-new-drug-substances-drug-products-scientific-guideline.

[13] De Lourdes Mata-Bilbao M., Andrés-Lacueva C., Roura E., Jáuregui O., Torre C., Lamuela-Raventós R.M. (2007). A New LC/MS/MS Rapid and Sensitive Method for the Determination of Green Tea Catechins and Their Metabolites in Biological Samples. J. Agric. Food Chem..

[14] Al-Duais M., Müller L., Böhm V., Jetschke G. (2009). Antioxidant Capacity and Total Phenolics of Cyphostemma Digitatum before and after Processing: Use of Different Assays. Eur. Food Res. Technol..

[15] Dotsikas Y., Loukas Y.L. (2003). Efficient Determination and Evaluation of Model Cyclodextrin Complex Binding Constants by Electrospray Mass Spectrometry. J. Am. Soc. Mass Spectrom..

[16] Kuhnert N., Clifford M.N., Müller A. (2010). Oxidative Cascade Reactions Yielding Polyhydroxy-Theaflavins and Theacitrins in the Formation of Black Tea Thearubigins: Evidence by Tandem LC-MS. Food Funct..

[17] Kuhnert N. (2010). Unraveling the Structure of the Black Tea Thearubigins. Arch. Biochem. Biophys..

[18] United States Pharmacopeia (2022). Dietary Supplement Monographs, Powdered Decaffeinated Green Tea Extract. USP-NF. Rockville, MD: United States Pharmacopeia. https://doi.usp.org/USPNF/USPNF_M2500_06_01.html.

[19] Guo Q., Zhao B., Shen S., Hou J., Hu J., Xin W. (1999). ESR Study on the Structure–Antioxidant Activity Relationship of Tea Catechins and Their Epimers. Biochim. Biophys. Acta (BBA)—Gen. Subj..

[20] Wang R., Zhou W., Jiang X. (2008). Reaction Kinetics of Degradation and Epimerization of Epigallocatechin Gallate (EGCG) in Aqueous System over a Wide Temperature Range. J. Agric. Food Chem..

[21] Suzuki M., Sano M., Yoshida R., Degawa M., Miyase T., Maeda-Yamamoto M. (2003). Epimerization of Tea Catechins and O-Methylated Derivatives of (−)-Epigallocatechin-3-*O*-Gallate: Relationship between Epimerization and Chemical Structure. J. Agric. Food Chem..

[22] Zhong Y., Ma C.-M., Shahidi F. (2012). Antioxidant and Antiviral Activities of Lipophilic Epigallocatechin Gallate (EGCG) Derivatives. J. Funct. Foods.

[23] Cai Z.-Y., Li X.-M., Liang J.-P., Xiang L.-P., Wang K.-R., Shi Y.-L., Yang R., Shi M., Ye J.-H., Lu J.-L. (2018). Bioavailability of Tea Catechins and Its Improvement. Molecules.

[24] Hong J., Lambert J.D., Lee S.-H., Sinko P.J., Yang C.S. (2003). Involvement of Multidrug Resistance-Associated Proteins in Regulating Cellular Levels of (−)-Epigallocatechin-3-Gallate and Its Methyl Metabolites. Biochem. Biophys. Res. Commun..

[25] Jodoin J., Demeule M., Béliveau R. (2002). Inhibition of the Multidrug Resistance P-Glycoprotein Activity by Green Tea Polyphenols. Biochim. Biophys. Acta (BBA)—Mol. Cell Res..

[26] Akazawa T., Uchida Y., Miyauchi E., Tachikawa M., Ohtsuki S., Terasaki T. (2018). High Expression of UGT1A1/1A6 in Monkey Small Intestine: Comparison of Protein Expression Levels of Cytochromes P450, UDP-Glucuronosyltransferases, and Transporters in Small Intestine of Cynomolgus Monkey and Human. Mol. Pharm..

[27] Savjani K.T., Gajjar A.K., Savjani J.K. (2012). Drug Solubility: Importance and Enhancement Techniques. ISRN Pharm..

