# Supplementary material for: 3D-Printed, Liquid-Filled Capsules of Concentrated and Stabilized Polyphenol Epigallocatechin Gallate, Developed in a Clinical Trial
Source: Antioxidants (Basel). 2023 Feb 9;12(2):424. doi: 10.3390/antiox12020424 (PMC9952645; doi:10.3390/antiox12020424)
Supplement: Supplementary file 1 [file antioxidants-12-00424-s001.zip › antioxidants-2194293-supplementary.pdf]

Supplementary information to:

3D PRINTED LIQUID-FILLED CAPSULES OF CONCENTRATED AND STABILIZED POLYPHENOL EPIGALLOCATECHINE GALLATE, DEVELOPED IN A CLINICAL TRIAL

Philippe-Henri Secretan[1,*], Victoire Vieillard[2], Olivier Thirion[2], Maxime Annereau[1,3], Hassane Sadou Yayé[4], Alain Astier[2], Muriel Paul[2,5], Thibaud Damy[6], Bernard Do[1,2]

1 Université Paris-Saclay, Matériaux et santé, 92296, Châtenay-Malabry, France.

2 Department of Pharmacy, Henri Mondor Hospital, AP-HP, Créteil, France

3 Clinical Pharmacy Department, Gustave Roussy Cancer Campus, France

4 Department of Pharmacy, Hôpitaux Universitaires Pitié-Salpêtrière, AP-HP, 75013 Paris, France

5 EpidermE, Univ Paris Est Creteil, 94010 Creteil, France

6 Department of Cardiology, Henri Mondor Hospital, AP-HP, Créteil, France

*corresponding author; email: philippe-henri.secretan@universite-paris-saclay.fr

Table of contents

List of tables 2

1. Stress testing protocol 3
2. Long-term stability studies 3
3. Chromatographic conditions of the stability study 5
4. Analysis of 'USP powdered decaffeinated green tea extract' reference standard 5

List of tables

Table S1: Stress conditions to assess EGCG stability 3
Table S2: Sampling and storage protocol for primary batch studies 3
Table S3: Gradient conditions 5

List of figures

Figure S1 : Chromatogram from analysis of 'USP powdered decaffeinated green tea extract' reference standard 5

1. Stress testing protocol

Table S1: Stress conditions to assess EGCG stability

Stress condition	Description	Exposure duration
Basic condition	Dilution of samples to be tested at 1/2 in a 0.1 mol/L NaOH solution.	Up to 6 days
Photolysis condition	The source used produces an output similar to the D65/ID65 emission standard. These irradiation conditions provide an overall illumination of not less than 1.2 million lux hours.	Up to 6 days
Thermolysis condition	70 °C	Up to 6 days

2. Long-term stability studies

The tested batch has been stored in accordance with ICH (The International Council for Harmonisation of Technical Requirements for Pharmaceuticals for Human Use) guideline Q1A(R2). Details of the sampling time points for each condition are presented in Table S2.

Table S2: Sampling and storage protocol for primary batch studies

Time (months)	Storage conditions	
	25°C/60%RH	40°C/75%RH
1	+	+
2	+	+
3	+	+
4	+	+
5	+	+
6	+	+
9	+	+
12	+	+
18	+	-

3. Chromatographic conditions of the stability study

The assay of EGCG was achieved by comparing the response of a sample solution with the response of EGCG reference standard solution prepared at a similar nominal concentration and analysed in the same way.

Separations were performed using column of Interchim® VKR5 C18 (250 x 4.6.0 mm, 5 µm particle size). Acetonitrile, acetic acid and pure water were used to prepare mobile phase A (5/95/0.07 v/v/v Acetonitrile/Water/Acetic acid) and mobile phase B (50/50/0.05 v/v/v Acetonitrile/Water/Acetic acid). Injection volume, flow rate and detection wavelength were set at 20µL, 1ml.min-1 and 275 nm. The gradient conditions are reported in Table S3.

Table S3: Gradient conditions

Time (min)	A (%)	B (%)
0	90	10
10	80	20
16	60	40
20	50	50
25	50	50
27	60	40
30	90	10
33	90	10

4. Analysis of 'USP powdered decaffeinated green tea extract' reference standard

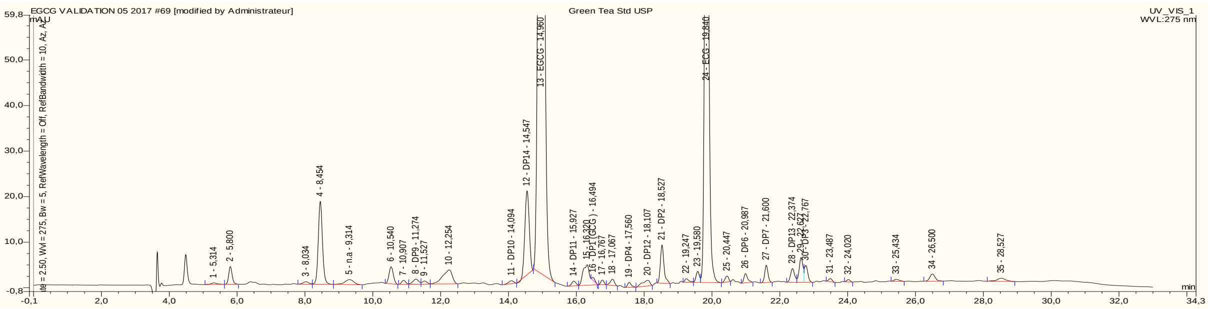

Figure S1 : Chromatogram from analysis of 'USP powdered decaffeinated green tea extract' reference standard
